# The genome of *Chenopodium pallidicaule*: An emerging Andean super grain

**DOI:** 10.1002/aps3.11300

**Published:** 2019-11-08

**Authors:** Hayley Mangelson, David E. Jarvis, Patricia Mollinedo, Oscar M. Rollano‐Penaloza, Valeria D. Palma‐Encinas, Luz Rayda Gomez‐Pando, Eric N. Jellen, Peter J. Maughan

**Affiliations:** ^1^ Department of Plant and Wildlife Sciences Brigham Young University 5144 LSB Provo Utah 84602 USA; ^2^ Institute of Natural Product Research Universidad Mayor de San Andrés La Paz Bolivia; ^3^ Departamento de Fitotecnia Facultad de Agronomía Universidad Nacional Agraria de La Molina La Molina Peru

**Keywords:** Amaranthaceae, Andean crops, *Chenopodium pallidicaule*, Hi‐C, proximity‐guided assembly

## Abstract

**Premise:**

Cañahua is a semi‐domesticated crop grown in high‐altitude regions of the Andes. It is an A‐genome diploid (2*n* = 2*x* = 18) relative of the allotetraploid (AABB) *Chenopodium quinoa* and shares many of its nutritional benefits. Cañahua seed contains a complete protein, a low glycemic index, and offers a wide variety of nutritionally important vitamins and minerals.

**Methods:**

The reference assembly was developed using a combination of short‐ and long‐read sequencing techniques, including multiple rounds of Hi‐C–based proximity‐guided assembly.

**Results:**

The final assembly of the ~363‐Mbp genome consists of 4633 scaffolds, with 96.6% of the assembly contained in nine scaffolds representing the nine haploid chromosomes of the species. Repetitive element analysis classified 52.3% of the assembly as repetitive, with the most common repeat identified as long terminal repeat retrotransposons. MAKER annotation of the final assembly yielded 22,832 putative gene models.

**Discussion:**

When compared with quinoa, strong patterns of synteny support the hypothesis that cañahua is a close A‐genome diploid relative, and thus potentially a simplified model diploid species for genetic analysis and improvement of quinoa. Resequencing and phylogenetic analysis of a diversity panel of cañahua accessions suggests that coordinated efforts are needed to enhance genetic diversity conservation within ex situ germplasm collections.

*Chenopodium pallidicaule* Aellen (also known as cañahua) is a species of the goosefoot family (Amaranthaceae) related to the increasingly popular seed crop, quinoa (*C. quinoa* Willd). Gade (1970) noted that cañahua is a partially domesticated crop that provides food security to many subsistence farmers across the Andean Altiplano—the high plateau situated at 3500–4200 m above sea level between the western and eastern Andean Cordilleras of west‐central South America. Cultivation of cañahua dates back more than 7000 years when it was a staple crop in ancient Incan and pre‐Incan societies. It has several common names in native languages, including *cañahua*, *cañigua*, *cañihua*, *cañawa*, and *kañiwa* (Gade, 1970). After the Spanish Conquest, cultivation was likely discouraged in colonial society due to its association with indigenous cultures (Ruas et al., 1999). Although it never completely regained its former status, subsistence farmers across the Andean region continue to grow cañahua due to its tolerance to abiotic stress (i.e., frost, drought, and salinity) in addition to its high nutritional quality. Despite the increasing popularity of its close relative quinoa, cañahua remains practically unknown and underutilized as a food resource (Rastrelli et al., 1996) outside of the Andes.

Cañahua has a unique nutritional profile that is ideal for human consumption in areas where protein is limited. Its seed contains 15–18% protein, with a complete set of essential amino acids, including 5–6% lysine, which is typically limiting in monocotyledonous grain crops (Penarrieta et al., 2008). In addition to high‐quality protein, cañahua offers a wide variety of other health‐promoting compounds, including antioxidants, phenols, and flavonoids (Repo‐Carrasco‐Valencia et al., 2010). Cañahua seeds contain vanillic acid, a phenolic compound which acts as a flavor enhancer and lends a pleasant taste to cañahua, particularly when ground and toasted as a flour called cañihuaco (Penarrieta et al., 2008). With a poverty rate of nearly 50% in the rural highlands of the Altiplano, cañahua represents an incredibly important resource in the prevention of poverty‐induced malnutrition and in improving food security throughout the region (Repo‐Carrasco et al., 2003).

Gade (1970) noted nearly half a century ago that the continued presence of cañahua in the Altiplano will depend on its genetic transformation into a more efficient crop. Agronomic issues that have prevented more extensive cultivation of cañahua include non‐uniform seed ripening and small seed size that make harvesting and processing of the seed difficult (Mujica, 1994). Despite its unique agronomic and nutritional qualities, very few of the genetic resources needed to accelerate the improvement of cañahua have been investigated. Ruas et al. (1999) published a phylogenetic study of 19 *Chenopodium* L. species based on RAPD markers, including two cañahua accessions that were found to be nearly identical. Vargas et al. (2011) developed the first microsatellite markers for cañahua, including 34 polymorphic markers, exhibiting a total of 154 different alleles. A phylogeny of 43 cañahua accessions showed clear distinctions between wild and cultivated lines, including a distinct subclade of erect morphotypes. Kolano et al. (2011) cytologically characterized the genome size and rDNA loci of cañahua. Their findings predicted a 2C value for the cañahua genome of 0.886 ± 0.034 pg (~433 Mbp per haploid genome) with a single copy of both 35S (subterminal) and 5S (interstitial) rDNA loci.

As a part of the genome analysis of quinoa, Jarvis et al. (2017) reported a draft assembly of the cañahua genome (accession PI 478407). Quinoa is an allotetraploid (2*n* = 4*x* = 36), presumably resulting from a relatively recent (3.3–6.3 mya) polyploidization event between North American and Eurasian diploids representing the A and B subgenomes of modern quinoa, respectively (Štorchová et al., 2015). Although cañahua is not believed to be the direct A‐genome donor of quinoa, it is a related A‐genome diploid. The draft genome reported by Jarvis et al. (2017) was based solely on Illumina short reads and was thus highly fragmented, consisting of 3015 scaffolds and spanning a total length of 337 Mbp, with an N50 of 356 kbp. Here we report the use of PacBio long reads and Hi‐C–based proximity‐guided assembly to develop a reference‐quality, chromosome‐scale assembly of cañahua. The genome was fully annotated using a deeply sequenced transcriptome developed from six combinations of tissue types and abiotic stresses. Additionally, genetic diversity within the species was characterized with a panel of 30 cultivated and wild cañahua varieties.

## METHODS

### Plant material

The cañahua accession PI 478407 was used to develop the reference assembly. It was originally collected in 1981 at the Instituto Boliviano de Tecnologia, Patacamaya, Bolivia, and is freely available from the United States Department of Agriculture (USDA; Ames, Iowa, USA; https://npgsweb.ars-grin.gov/). The diversity panel consisted of 30 accessions from three germplasm collections: specifically, eight cañahua varieties from the USDA collection (https://npgsweb.ars-grin.gov/), one landrace and two wild accessions from the Universidad Nacional Agraria La Molina (UNALM; Lima, Peru), and 21 accessions from Universidad Major de San Andrés (UMSA; La Paz, Bolivia). A complete list of all plant materials used is provided in Table 1.

**Table 1:** Passport and sequence archive information for plant materials used. Raw sequencing data for each accession are deposited in the Sequence Read Archive (SRA) at the National Center for Biotechnology Information (NCBI).

Name	Collection^a^	Accession ID	Collection location	Altitude (m a.s.l.)	Sequencing technology	SRA ID^b^
WGS reference information						
PI 478407	USDA	PI 478407	−17.2333, −67.9166	3800	PacBio	SRR9661228
PI 478407	USDA	PI 478407	−17.2333, −67.9166	3800	Hi‐C (Illumina)	SRR9661229
PI 478407	USDA	PI 478407	−17.2333, −67.9166	3800	WGS (Illumina)	SRR4425239^c^
PI 478407	USDA	PI 478407	−17.2333, −67.9166	3800	RNA‐Seq	SRR4425240–SRR4425243^c^
Diversity panel information						
P1	UNALM	BYU 1780	−15.6967, −70.20510	3830	WGS (Illumina)	SRR9620980
P2	UNALM	BYU 1781	−15.7268, −70.23560	3838	WGS (Illumina)	SRR9640749
P4	UNALM	BYU 1785	−15.7693, −70.27050	3860	WGS (Illumina)	SRR9640748
U7	USDA	PI 510525	−16.3628, −69.2765	NA	WGS (Illumina)	SRR9640742
U8	USDA	PI 510526	−16.2833, −69.2833	NA	WGS (Illumina)	SRR9640741
U9	USDA	PI 510527	−16.0000, −69.7833	3810	WGS (Illumina)	SRR9640740
U12	USDA	PI 510530	−16.4500, −70.2333	NA	WGS (Illumina)	SRR9640747
U13	USDA	PI 665279	−17.2333, −67.9166	3700	WGS (Illumina)	SRR9640746
U14	USDA	PI 665280	−17.2333, −67.9166	3700	WGS (Illumina)	SRR9640745
U15	USDA	PI 665281	−17.2333, −67.9166	3700	WGS (Illumina)	SRR9640744
U16	USDA	PI 665282	−17.2333, −67.9166	3700	WGS (Illumina)	SRR9640743
B17	UMSA	Bol‐1.1	−15.7472, −68.8091	3845	WGS (Illumina)	SRR9640755
B18	UMSA	Bol‐3.1	−16.5344, −68.0622	3445	WGS (Illumina)	SRR9640754
B20	UMSA	Bol‐19.1	−17.8241, −67.7702	3721	WGS (Illumina)	SRR9640757
B21	UMSA	Bol‐20.123	−17.7850, −68.1447	4025	WGS (Illumina)	SRR9640756
B22	UMSA	Bol‐21.123	−17.6483, −67.2072	3777	WGS (Illumina)	SRR9640751
B23	UMSA	Bol‐22.123	−18.2166, −67.0333	3707	WGS (Illumina)	SRR9640750
B24	UMSA	Bol‐23.123	−16.5344, −68.0622	3445	WGS (Illumina)	SRR9640753
B25	UMSA	Bol‐24.123	−16.6740, −68.3183	3900	WGS (Illumina)	SRR9640752
B26	UMSA	Bol‐25.123	−16.5344, −68.0622	3445	WGS (Illumina)	SRR9640759
B27	UMSA	Bol‐26.123	−16.5344, −68.0622	3445	WGS (Illumina)	SRR9640758
B28	UMSA	Bol‐28.123	−16.6740, −68.3183	3900	WGS (Illumina)	SRR9640732
B29	UMSA	Bol‐29.123	−16.5344, −68.0622	3445	WGS (Illumina)	SRR9640733
B30	UMSA	Bol‐30.123	−17.2500, −67.9166	3800	WGS (Illumina)	SRR9640734
B31	UMSA	Bol‐4.3	−16.6740, −68.3183	3900	WGS (Illumina)	SRR9640735
B32	UMSA	Bol‐6.2	−16.6740, −68.3183	3900	WGS (Illumina)	SRR9640736
B33	UMSA	Bol‐7.1	−16.6740, −68.3183	3900	WGS (Illumina)	SRR9640737
B34	UMSA	Bol‐8.1	−16.6740, −68.3183	3900	WGS (Illumina)	SRR9640738
B35	UMSA	Bol‐13.3	−16.6740, −68.3183	3900	WGS (Illumina)	SRR9640739
B36	UMSA	Bol‐27.123	−16.6740, −68.3183	3900	WGS (Illumina)	SRR9640731

Note

m a.s.l. = meters above sea level; NA = not available.

a Germplasm collection center. USDA = United States Department of Agriculture, Ames, Iowa, USA; UNALM = Universidad Nacional Agraria La Molina, Lima, Peru; UMSA = Universidad Major de San Andrés La Paz, Bolivia; BYU = Brigham Young University, Provo, Utah, USA.

b Sequence Read Archive (SRA) identifier.

c Deposited in BioProject ID PRJNA326220. All other sequences are deposited in BioProject ID PRJNA552289.

### Whole genome assembly

In vivo Hi‐C and proximity‐guided assembly techniques were used to improve the previously published short‐read draft assembly reported by Jarvis et al. (2017), referred to hereafter as the ALLPATHS‐LG short‐read assembly (ASRA). Fresh leaf tissue from a single, dark‐treated (72 h) 3‐week‐old plant that was derived directly from selfing of the original cañahua ‘PI 478407’ plant used by Jarvis et al. (2017) was sent to Phase Genomics (Seattle, Washington, USA) for in vivo Hi‐C–based proximity‐guided ligation and 80‐bp paired‐end sequencing followed by alignment to the ASRA assembly using BWA version 0.7 (Li and Durbin, 2010). Only reads that aligned uniquely to the scaffolds were retained. Proximo, a proximity‐guided assembly method based on the ligating adjacent chromatin enables scaffolding in situ (LACHESIS) assembler (Burton et al., 2013), was used to cluster, order, and orient scaffolds from the ASRA assembly, producing the first proximity‐guided assembly (PGA1). Following the development of PGA1, long reads were used for gap‐filling. High‐molecular‐weight DNA was extracted from leaf tissue of a single, 72‐h dark‐treated cañahua (PI 478407) plant using the QIAGEN Genomic‐tip 500/G Kit (Hilden, Germany). Single‐molecule, real‐time sequencing using the PacBio Sequel platform (Menlo Park, California, USA) was performed at the Brigham Young University DNA Sequencing Center (Provo, Utah, USA). The PBJelly2 pipeline from PBSuite version 15.8.24 (English et al., 2012) was used to align the long reads to PGA1 in order to gap‐fill the assembly. Arrow version 0.22.0 (Chin et al., 2013) and Pilon version 1.22 (Walker et al., 2014) were used for genome polishing with the previously described PacBio long reads and Illumina paired‐end reads, respectively. This gap‐filled and polished assembly is henceforth referred to as PGA1.5. To correct for possible errors introduced by low PacBio read coverage and relaxed PBJelly2 parameters, a contig‐breaking tool, Polar Star (https://github.com/phasegenomics/polar_star), was employed. Polar Star aligns long reads to an assembly, then calculates the read depth at each base. Read depth is smoothed in a 100‐bp sliding window, then regions of high, low, and normal read depth are merged. These classifications are made based on the read depth distribution. Low‐read‐depth outliers are identified, and the assembly is broken at each such location. Following Polar Star, PGA1.5 underwent a second de novo, proximity‐guided assembly. Assembly errors (inversions and rearrangements) were identified and adjusted manually using Juicebox version 1.9.8 (Durand et al., 2016). The result was a chromosome‐scale, polished assembly referred to as PGA2 (Appendix S1).

### Transcriptome assembly, gene annotation, and repeat modeling

RNA‐Seq data was generated on the Illumina Hi‐Seq platform from cañahua (PI 478407) leaf, root, inflorescence, and apical meristem tissues grown in both non‐stressed and salt‐stressed conditions, as detailed by Jarvis et al. (2017). The reads were trimmed using Trimmomatic version 0.32 (Bolger et al., 2014) to remove Illumina adapters and trailing bases with a quality score below 20, then aligned to the PGA2 reference using HiSat2 version 2.0.4 (Kim et al., 2015; Pertea et al., 2016) with default parameters except the max intron length was set to 50,000 bp. After alignment, the resulting sequence alignment map (SAM) file was sorted and indexed using SAMtools version 1.6 (Li et al., 2009) and assembled into putative transcripts using StringTie version 1.3.4 (Pertea et al., 2016). Whole‐genome annotation of the PGA2 assembly was performed by MAKER version 2.31.10 (Holt and Yandell, 2011) using the cañahua transcriptome as expressed sequence tag (EST) evidence, the uniprot_sprot database (downloaded 25 September 2018) and quinoa protein sequences (Jarvis et al., 2017) as protein homology evidence, and the consensi.fa.classified output from RepeatModeler for soft repeat masking. Gene prediction models included an Augustus model for cañahua produced by Benchmarking Universal Single‐Copy Orthologs (BUSCO) version 3.0.2 (Simao et al., 2015) and the *Arabidopsis thaliana* SNAP HMM file (Korf, 2004) for gene prediction. BUSCO version 3.0.2 assessed the completeness of the assembly and annotation using the embryophyta odb10 data set. RepeatModeler version 1.0.11 and RepeatMasker version 4.0.7 (Smit et al., 2013–2015) were used to identify and classify repetitive elements in the final (PGA2) assembly relative to Repbase‐derived RepeatMasker libraries version 20181026 (Bao et al., 2015).

### Chloroplast genome assembly and annotation

A reference‐guided assembly of the cañahua chloroplast genome was constructed by the Assembly by Reduced Complexity (ARC) assembler version 1.1.4 (Hunter et al., 2015) using a subset of six million whole‐genome, paired‐end Illumina reads with the quinoa chloroplast genome (Maughan et al., 2019) as a target. The ARC algorithm uses Bowtie2 (Langmead and Salzberg, 2012) with relaxed parameters to map reads against targets, extract mapped reads from each target, and assemble mapped reads using the SPAdes assembler (Bankevich et al., 2012). The targets are then replaced with newly assembled contigs, and the process is iterated for a predetermined number of cycles or until no additional reads can be incorporated. The ARC pipeline extended the assembled cañahua chloroplast contigs through four (numcycles = 4) successive rounds of mapping and re‐assembly. Because chloroplast read depth should be significantly higher than nuclear genome read depth, only assembled contigs with read depth >50× coverage were selected for further assembly. Pacific Biosciences long reads (>15 kbp; *n* = 246,847) were used to fill gaps between contigs using PBJelly2, a subprogram from PBSuite version 15.8.24 (English et al., 2012). A circularized contig representing the complete plastid genome was constructed using the circularize tool from Geneious (version 11.1.5; https://www.geneious.com/), then the assembly was polished with the same six million paired‐end Illumina reads as used in the initial assembly. Annotation of the cañahua chloroplast genome was performed using GeSeq version 1.65 (Tillich et al., 2017) with the quinoa chloroplast annotation (Maughan et al., 2019) and the Max Planck Institute of Molecular Plant Physiology (MPI‐MP) chloroplast database as references. ARAGORN version 1.2.3 and HMMER profile searches were enabled, the latter using the embryophyta chloroplast (CDS + rRNA) database. Comparison to the quinoa plastid genome was performed by the nucmer tool from MUMmer version 4.0beta (Marcais et al., 2018) followed by MUMmerplot with all default parameters.

### Resequencing and single‐nucleotide polymorphism discovery

DNA was extracted from single plants for each of 30 cañahua accessions using a cetyltrimethylammonium bromide (CTAB) extraction method as described by Doyle and Doyle (1987). Samples were sent to Novogene (San Diego, California, USA) for whole‐genome Illumina HiSeq (150‐bp paired‐end) sequencing from 500‐bp insert libraries. Trimmomatic version 0.32 (Bolger et al., 2014) was used to remove Illumina adapters and trailing bases with a quality score below 20 or average per‐base quality of 20 over a four‐nucleotide sliding window. Reads from each accession were aligned to PGA2 using BWA‐MEM version 0.7.17 (Li, 2013) to produce SAM files that were converted to binary alignment map (BAM) format, sorted, and indexed using SAMtools version 1.9 (Li et al., 2009). The BAM files were used as input for InterSnp, a subprogram of the BamBam version 1.4 pipeline (Page et al., 2014), for single‐nucleotide polymorphism (SNP) genotyping. SNPhylo version 20160204 (Lee et al., 2014) used the HapMap output files produced by InterSnp to filter and remove SNPs with >10% missing data and minor allele frequency <5%. SNPhylo also filters SNP data sets using linkage disequilibrium (LD) estimates (SNPs with LD < 40% are removed) prior to building bootstrapped (*n* = 1000) phylogenies based on MUSCLE (Edgar, 2004) sequence alignments. The resulting tree was visualized in FigTree version 1.4.3 (http://tree.bio.ed.ac.uk/software/figtree). Population structure was evaluated using Structure version 2.3.4 (Pritchard et al., 2000) with a range of *K* = 1 through *K* = 5 from a 1000 SNP subset of the InterSnp output. ArcMap version 10.3.1 (ArcGIS Desktop, release 10; Esri, Redlands, California, USA) mapping software was used to map the geographic locations of the source materials. The clustering partitions produced by Structure were used to construct a pie chart representing the allelic composition of each mapped individual.

### Genome comparison

Comparisons of coding sequences for quinoa (*C. quinoa*; CoGe id53523), beet (*Beta vulgaris* L.; CoGe id37197; Funk et al., 2018), and amaranth (*Amaranthus hypochondriacus* L.; CoGe id34733; Lightfoot et al., 2017) were made using the CoGe SynMap tool (https://genomevolution.org/coge/) applying the Last algorithm with the recommended DAGChainer option (relative gene order) and Merge syntenic blocks option (quota align merge). The syntenic depth was set to quota align merge, at a ratio of coverage depth of 1 : 1 (beet) or 1 : 2 (quinoa, amaranth). The DAGchainer (Haas et al., 2004) output files were used as input for the MCScanX (Wang et al., 2012) and VGSC toolkit (Xu et al., 2016) for data visualization.

## RESULTS

### Whole genome assembly

The previous draft assembly of PI 478407 reported in Jarvis et al. (2017) was based solely on Illumina short reads assembled using the ALLPATHS‐LG assembler (Gnerre et al., 2011). Although this was an excellent draft assembly, the lack of long‐jump libraries (i.e., fosmid) or bacterial artificial chromosome (BAC)‐end sequencing resulted in a highly fragmented assembly. The ASRA assembly consisted of 8982 contigs in 3013 scaffolds with a contig and scaffold N50 of 84 kbp and 357 kbp, respectively, spanning a total length of 337 Mbp (Table 3). To improve the ASRA, 179 million Hi‐C–based paired‐end reads were generated and used to scaffold the ASRA using the Proximo pipeline (Phase Genomics). Seventy‐nine percent (2392) of the ASRA scaffolds were clustered into nine pseudomolecules, corresponding to the nine haploid chromosomes of cañahua (2*n* = 2*x* = 18; Appendix S1), producing a substantially improved proximity‐guided assembly (PGA1). The number of scaffolds clustered to specific chromosomes ranged from 203 to 317, and the length of the assembled chromosomes ranged from 31.3 to 40.4 Mbp. The PGA1 scaffolds contained 95.3% of the total sequence length (99.7% excluding N gaps) with an N50 and L50 of 35.6 Mbp and 5, respectively (Table 3). Ns occupied 12.3 Mbp (4%) of the assembly, with an average of 1047 gaps (20 or more contiguous Ns) per scaffold. The unincorporated scaffolds (621) were small, representing <5% of the total sequence length of the ASRA, with a mean scaffold size of 25.8 kbp, making them much more difficult to incorporate accurately into chromosomes.

**Table 3:** Assembly statistics for the ASRA, PGA1, PGA1.5, and PGA2 assemblies.

Assembly statistic	ASRA	PGA1	PGA1.5	PGA2
Assembly size (Mbp)	337	337	363	363
No. of scaffolds	3015	623	591	4633
Scaffold N50 size (Mbp)	0.357	35.6	37.8	38.1
Scaffold L50 count	243	5	5	5
Longest scaffold (Mbp)	2.9	40.4	43.2	45.5
No. of contigs	8984	8984	2580	8210
Contig N50 size (Mbp)	0.083	0.083	0.516	0.236
Contig L50 count	1096	1096	168	401
% missing bases	2.5	2.6	0.2	0.1
Assembly size (Mbp) in top 9 scaffolds	20	321	344	350
Assembly % in top 9 scaffolds	5.8	95.4	94.8	96.5

Note

ASRA = ALLPATHS‐LG Short‐Read Assembly; PGA1 = Proximity‐Guided Assembly 1; PGA1.5 = Proximity‐Guided Assembly 1.5; PGA2 = Proximity‐Guided Assembly 2.

PGA1 was further improved by applying a combination of gap‐filling and genome‐polishing techniques. To close gaps, 10.21 Gbp (1,101,202 reads) of PacBio long reads were generated with a mean read length of 9.3 kbp, providing 23.6× coverage of the cañahua genome. PacBio long reads were aligned to PGA1 using PBJelly2 (English et al., 2012), closing 75% of existing N gaps. Due to potential errors introduced into gaps because of the inherent high error rate of PacBio reads, the assembly quality was improved using two genome‐polishing tools: Arrow (Chin et al., 2013), which produces consensus‐quality assemblies from PacBio sequences, followed by Pilon (Walker et al., 2014), which performs a similar function but takes advantage of the significantly lower error rate of Illumina reads to improve the consensus assembly. These polishing steps made changes at 593,821 positions, representing <0.165% of PGA1. The resulting assembly, PGA1.5, had a total size of 363 Mbp, an approximately 7.7% increase from the ASRA. The scaffold N50 of PGA1.5 increased slightly to 37.8 Mbp, while the number of gaps decreased dramatically from 8013 to 2007, which is also reflected in a 10‐fold decrease in the number of Ns in the assembly (4% to 0.2%; Table 3).

A second round of proximity‐guided assembly using PGA1.5 improved the chromosome‐scale assembly. Polar Star, which aggressively breaks contigs at low PacBio depth locations based on deviation from mean depth, introduced 5241 breaks that were then tested for rescaffolding using Hi‐C–based proximity‐guided assembly. This acts as a check on the error‐prone PacBio reads and low coverage depth used in the gap‐filling process. The result is a dramatically improved proximity‐guided assembly, evident by the consistent pattern of Hi‐C crosslink density along chromosomes and the resolution of erroneous inversions and rearrangements. The final assembly (PGA2) spans 362.5 Mbp, has a scaffold N50 and L50 of 38.1 Mbp and 5, respectively, with <0.1% of the assembled sequence found in 3586 gaps. Eighty‐four percent of the estimated genome size is represented; the remaining 16% is likely composed of repetitive sequence that has collapsed in regions such as centromeres and telomeres due to the use of short reads for the initial assembly. The nine chromosomes contain 96.7% of the total sequence length (99.9% excluding N gaps), ranging in size from 33.5 Mbp to 45.4 Mbp (Appendix S2).

### Repeat identification and genome annotation

**Figure 1:**
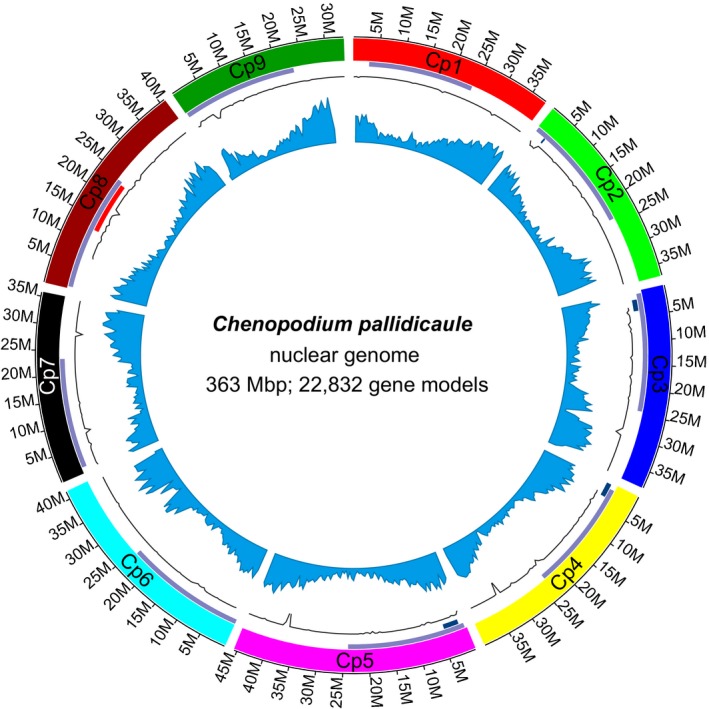
Genome annotation overview. An overview of gene and repetitive element annotations in the *Chenopodium pallidicaule* genome. Track 1: chromosome names and sizes; Track 2: frequency of pericentromeric 12‐13P repetitive elements (purple); Track 3: frequency of 18‐24J repetitive element (blue) and the 5S rRNA locus (red); Track 4: frequency of canonical telomeric repeat; Track 5: gene density.

RepeatModeler and RepeatMasker were employed to identify and classify repetitive elements in the cañahua genome. Fifty‐three percent (191 Mbp) of the cañahua genome was classified as repetitive, with an additional 1.9% (7 Mbp) classified as low complexity (satellites, simple repeats, and small RNAs). A total of 129 Mbp (35.5%) was identified as retrotransposons or DNA elements, with an additional 61 Mbp (16.8%) classified as unknown elements. The most common elements identified were long terminal repeat (LTR) retrotransposons, specifically *copia*‐like and *gypsy*‐like elements, which spanned 67 Mbp (27.1%) of the genome (Appendix S3). The large fraction of unknown elements is not surprising given that the only published studies of repetitive elements in the *Chenopodium* genus have been limited to rDNA sequences (Maughan et al., 2006; Kolano et al., 2011) and two repetitive sequences, 18‐24J and 12‐13P, that were only recently characterized cytogenetically (Orzechowska et al., 2018). BLASTn was used to locate the 5S rDNA sequence and the two *Chenopodium* repetitive elements within the final assembly. Consistent with the findings of Kolano et al. (2011), the 5S rDNA sequence was found in a single genomic location in the centromeric region of chromosome Cp8 (Fig. 1). Orzechowska et al. (2018) previously reported that the 18‐24J repeat was almost exclusively found in the *Chenopodium* B‐genome, whereas 12‐13P was located at pericentric positions in both the A‐ and B‐genomes. BLASTn searches of the cañahua genome confirmed these observations, with 18‐24J identified in only 0.012% of the cañahua genome while the 12‐13P repetitive element, occupying 124.6 kbp (0.027%), was localized to putative centromeric regions on all nine chromosomes (Fig. 1).

A transcriptome assembly of cañahua was developed by sequencing RNA‐Seq libraries from six unique tissue and abiotic stress combinations. The resulting RNA‐Seq libraries generated 66.3 Gbp of data from 663,493,956 paired‐end reads with an average of 11.05 Gbp per library. Ninety‐eight percent (649,273,284) of the paired RNA‐Seq reads aligned to the final PGA2 assembly, with 97.9% of those read pairs aligning concordantly—suggestive of a high‐quality genome assembly. A Stringtie (Pertea et al., 2016) reconstruction of the cañahua transcriptome identified 255,893 features, including 214,170 exons in 41,723 primary and alternative transcripts with a mean transcript length of 2.19 kbp and an average of 28,246 features per chromosome.

The MAKER pipeline was used to annotate PGA2 using as evidence the cañahua transcriptome (described above) and, as alternative evidence, the transcripts and protein sequences for quinoa (Jarvis et al., 2017), as well as the complete uniprot_sprot database. A total of 22,832 gene models were annotated, which is slightly more than half of the 44,776 gene models annotated in the allotetraploid quinoa (Fig. 1). The average transcript length was 4.6 kbp, with the longest protein sequence spanning 4769 amino acids (annotation ID: CP013000), which is predicted to encode the large *sacsin*‐like gene found in many eukaryotes, including other Amaranthaceae species such as quinoa (XP_021735414), beet (XP_010688704), and spinach (XP_021846357). The mean annotation edit distance (AED), which is a quality measure combining values for sensitivity, specificity, and accuracy to give evidence of a high‐quality annotation, was 0.23. AED values <0.3 are indicative of high‐quality annotations (Holt and Yandell, 2011).

Completeness of the gene space was assessed using the BUSCO platform, which quantifies functional gene content using a large core set of highly conserved orthologous genes (COGs). Of the 1375 plant‐specific COGs in the embryophyta database, 1341 (97.5%) were identified in the cañahua genome as complete, with another nine (0.7%) COGs classified as fragmented (complete: 97.5% [single: 95.9%, duplicated: 1.6%], fragmented: 0.7%, missing: 1.8%). Relative to the MAKER de novo annotated proteins and transcripts, BUSCO identified 1260 (91.6%) and 1303 (94.8%) complete COGs, respectively. The discrepancies between the whole genome, protein, and transcript BUSCO findings may be attributed to the difference in gene annotation method between BUSCO and MAKER. Whereas BUSCO uses BLAST to identify known genes, MAKER uses an approach that requires sufficient evidence from a combination of protein, EST, and ab initio gene prediction inputs. The annotation could potentially be improved by further training of the input gene prediction model (Augustus, SNAP).

### Chloroplast genome reconstruction

**Figure 2:**
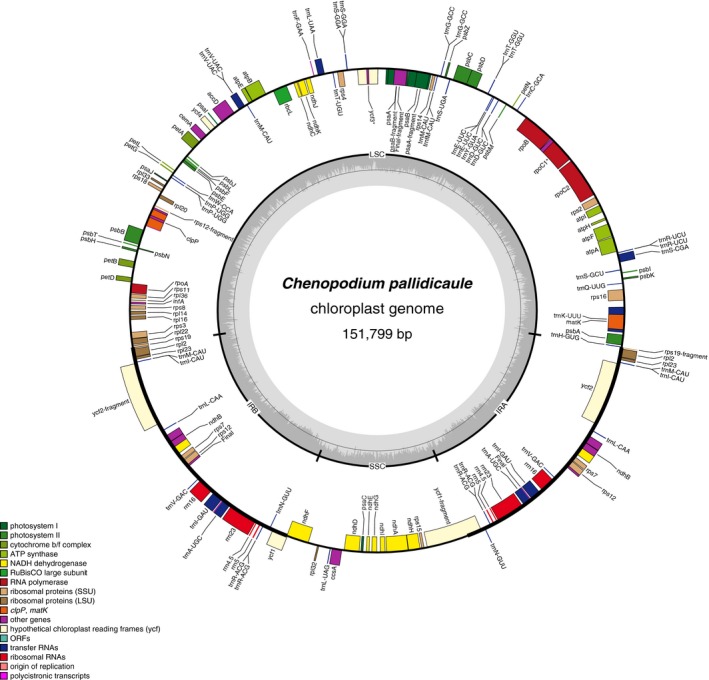
Chloroplast annotation overview. The outside track shows genes transcribed in a clockwise direction, the second track shows genes transcribed in a counterclockwise direction, and the inside track shows G/C content levels. Annotation reveals a quadripartite structure, including two copies of the inverted repeat (bolded line) dividing large and small single‐copy regions.

The cañahua chloroplast assembly spans 151,799 bp in a single circular molecule. Annotation of the chloroplast genome revealed a quadripartite structure, including two copies of an inverted repeat (IR) region separating large and small single‐copy regions. One hundred thirty‐two genes were identified, including 88 protein‐coding genes, 36 tRNA genes, and eight rRNA genes (Fig. 2). Twenty‐one genes were located in each IR, including a pseudogene previously characterized in other Amaranthaceae species as *rpl23* (Park et al., 2018; Maughan et al., 2019). With a length of 151,799 bp, the cañahua plastid genome is of a similar size to quinoa, which has been reported for multiple quinoa accessions ranging in size from 152,079–152,282 bp, with an average length of 152,134 bp (Hong et al., 2017; Maughan et al., 2019). Due to the lack of recombination in chloroplast genomes and the relatively recent allotetraploidization event leading to quinoa (3.3–6.3 mya; Jarvis et al., 2017), the high degree of similarity between the cañahua and quinoa chloroplasts supports the hypothesis that the maternal parent in the polyploidization event that led to modern quinoa was an A‐genome species (Maughan et al., 2019).

### Diversity panel resequencing

**Figure 3:**
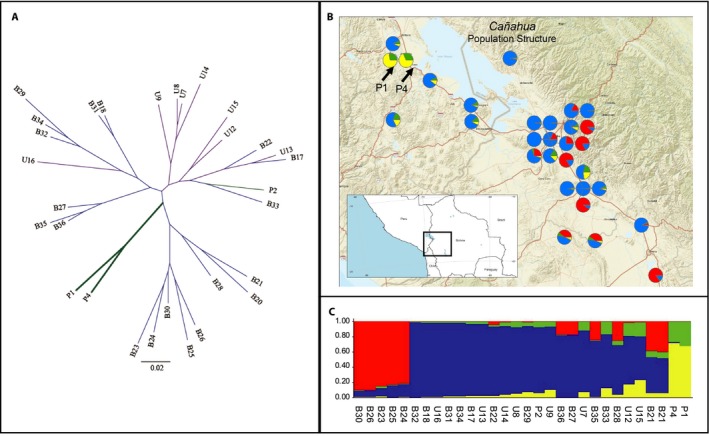
Diversity panel. (A) The unrooted tree was developed using 16,194 single‐nucleotide polymorphisms (SNPs) filtered to remove SNPs with >10% missing data, minor allele frequency <5%, and linkage disequilibrium <40%. Colors represent the collection source (purple = United States Department of Agriculture, green = Universidad Nacional Agraria La Molina, blue = Universidad Major de San Andrés La Paz), and bolded lines indicate wild accessions. (B) Geographic location (see Table 1 for passport information) combined with population structure information developed by Structure with *K* = 4. There is no significant correlation between collection site and genetic distance (*P* = 0.837). The wild *Chenopodium pallidicaule* accessions are identified with arrows. (C) Population structure and admixture in the diversity panel.

A diversity panel consisting of 30 varieties of cañahua, including 28 landrace varieties and two wild accessions, was sequenced to an average depth of 10.9× coverage (4.7 Gbp) per accession. After read alignment to the PGA2 final assembly, a total of 358,461 SNPs were identified in the diversity panel, which were then filtered to 16,194 SNPs, based on minor allele frequency, missing data, and linkage disequilibrium, with an average of 1799 SNPs per chromosome. Analysis of the consensus, 1000‐bootstrap phylogeny of the cañahua diversity panel suggests several major points of interest (Fig. 3A). First, the USDA collection of the species is limited to only two of three major groups, with the majority (seven out of eight accessions) on a single group, suggesting limited diversity within the USDA collection and highlighting the need for international collection efforts to preserve diversity within the species. Second, the Mantel test suggests that there is no correlation between collection site and genotype (Z = 11,296.22, *r* = −0.12326, and *P* = 0.837). This is likely due to a lack of good collection site data for many of the accessions. Indeed, eight of the accessions have as their passport data the latitude and longitude coordinates of the research facilities where they are curated instead of the coordinates of the original collection site (Fig. 3B, Table 1). Another potentially complicating issue is the well‐known cultural practice of seed trading among indigenous Andean societies that was an important part of agriculture in the pre‐Columbian Altiplano region for thousands of years (Vargas et al., 2011). Lastly, the two wild accessions (P1, P4) are found by themselves on a distinct clade within the phylogeny. A structure analysis (Pritchard et al., 2000) suggests that they are distinct from the landrace and cultivated accessions, showing little admixture with cultivated accessions, even though they were collected in close proximity to a cultivated type (P2, Fig. 3C). This finding agrees with those of Vargas et al. (2011) and is further evidence that the wild accessions may be useful sources of novel genetic diversity for improving cañahua.

### Genome comparison

**Figure 4:**
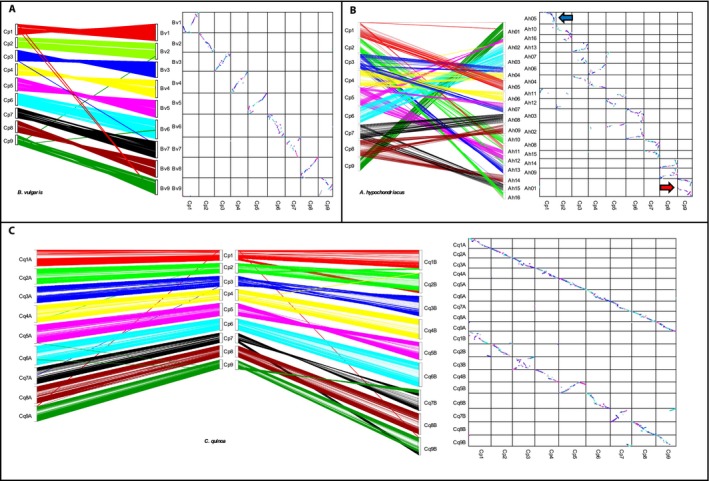
Genomic comparison of cañahua with beet, amaranth, and quinoa. Synteny dot plot (left) and dual syteny plots (right) show syntenic regions between cañahua and beet (A), amaranth (B), and quinoa (C) coding sequences. The dual synteny plot of the quinoa genome is divided into A‐ and B‐subgenomes with cañahua in the center. Increasing color intensity is associated with increasing homology in the dot plots. The arrows identify the chromosomal fusion (red) and loss (blue) in amaranth.

Syntenic relationships between cañahua and other Amaranthaceae species were explored using DAGChainer (Haas et al., 2004), which identifies colinear sets of homologous gene pairs (syntenic blocks) between genomes. The first species of the family with a reference‐quality chromosome‐scale assembly was beet (*B. vulgaris*; 2*n* = 2*x* = 18; Dohm et al., 2014). A genomic comparison between cañahua and beet identified 162 syntenic blocks with 11,659 colinear gene pairs (average of 72 genes/block) spanning 271 Mbp. As expected, given the relatively close ancestry of the species, the size (in base pairs) of the syntenic blocks between species was highly correlated (*R* ^2^ = 0.80). The large blocks of syntenic genes are suggestive of homologous relationships between the chromosomes of the two species (Fig. 4A). Indeed, homologous chromosome pairs can easily be identified for all cañahua and beet chromosomes: Cp1 = Bv1 (93% shared syntenic block sequence), Cp2 = Bv2 (100%), Cp3 = Bv3 (99%), Cp4 = Bv4 (100%), Cp5 = Bv5 (100%), Cp6 = Bv6 (100%), Cp7 = Bv7 (100%), Cp8 = Bv8 (100%), Cp9 = Bv9 (96%). To maintain the family naming convention, we have assigned the cañahua chromosomes with the same number as their beet homologs (i.e., Cp1 = Bv1, etc.).

Beet and cañahua are diploids that share a base chromosome number of *x* = 9, whereas the base number in *Amaranthus* is *x* = 8. Lightfoot et al. (2017) identified evidence of chromosome loss (the homoeolog of Ah5) and chromosome fusion (Ah1) in the amaranth genome that likely led to the observed base chromosome number reduction in the amaranths. Our comparison of cañahua to the amaranth genome identified 285 syntenic blocks with 13,200 colinear gene pairs. Although there was an increase in syntenic blocks identified in the cañahua–amaranth comparison, the number of genes per block dropped (46 genes/block) and was accompanied by a lower syntenic block size correlation (*R* ^2^ = 0.25). The decrease in block size and correlation is reflective of the more distant evolutionary relationship between these two species. Our analysis confirms the chromosome loss and fusion events in the amaranth genome. Indeed, the entirety of Cp9 aligns twice (end‐to‐end) with Ah1, and one homolog of Cp1 is largely missing (Fig. 4B). The synteny observed among the cañahua and amaranth chromosomes suggests several homoeologous relationships within the amaranth genome. For example, cañahua chromosome Cp6 is clearly homologous across the entirety of amaranth chromosomes Ah2 and Ah3. Indeed, of the 54 Mbp of amaranth sequence that is syntenic with Cp6, 48% (26 Mbp) is syntenic to Ah2 and 52% (28 Mbp) is syntenic to Ah3—clearly suggestive that Ah2 and Ah3 are homoeologs. Using the syntenic data from the cañahua–amaranth comparison and a simple majority rule (>75% syntenic sequence), we identify the following orthologous relationships: Cp1 = Ah5 (homoeolog loss), Cp2 = Ah10/Ah16, Cp4 = Ah6/Ah4, Cp6 = Ah3/Ah2, Cp7 = Ah8/Ah15, Cp8 = Ah14/Ah9, and Cp9 = Ah1/Ah1 (homoeolog fusion). Only one of the amaranth homoeologs of Cp3 is clearly identifiable in the data, specifically Ah13, with Ah7 likely the homoeolog, but obscured by a large translocation between Ah7 and Ah4. Similarly, the orthologous relationship with Cp5 likely involves Ah11 and Ah12 but is complicated by translocations with Ah2 (Fig. 4B, Appendix S4).

Comparison of cañahua to the quinoa genome identified 418 syntenic blocks with 23,410 colinear gene pairs (Appendix S5). When analyzed on a subgenome basis, cañahua had considerably more and gene‐dense syntenic blocks with subgenome A (13,073 gene pairs, 71.1 genes/block) relative to subgenome B (10,337 gene pairs, 46.2 genes/block; Appendix S5). The size of the sytenic blocks were also more highly conserved with the A subgenome relative to the B subgenome (*R* ^2^ = 0.82 and 0.35, respectively) as well as the total number of syntenic genes (Table 4), confirming that cañahua is representative of the A‐genome species in the genus. Although both the A and B subgenomes have maintained similar chromosomal structure, the A‐subgenome homoeologs in quinoa can be clearly identified via visual inspection of the syntenic sequence dot plots and are supported by the amount of syntenic bases shared (Fig. 4C, Appendix S4). All quinoa A chromosomes share a higher number of syntenic genes with cañahua than their B homoeologs, except for Cq4A and Cq4B where 1376 and 1416 syntenic genes were identified, respectively. However, the number of genes per syntenic block shared with Cp4 is higher in Cq4A (76.4) than for Cq4B (70.8), as is the total amount of syntenic bases shared with Cp4 (28.3 Mbp), which is suggestive that the assignment of Cq4A to the A‐subgenome is likely correct. This is even more significant considering that the B‐subgenome of quinoa is larger than the A‐subgenome (531 Mbp in the A‐subgenome and 670 Mbp in the B‐subgenome; Jarvis et al., 2017).

**Table 4:** Comparison of gene synteny, synonymous substitutions rate, and divergence since the last common ancestor relative to cañahua.

Metric	Amaranth	Beet	Quinoa A‐subgenome	Quinoa B‐subgenome
Total no. of genes^a^	45,947	45,334	43,663	44,638
Unique syntenic genes^b^	23,878	23,075	26,230	25,327
% of syntenic genes	52.0	50.9	60.1	56.7
Syntenic genes/block	46.3	71.9	71.1	46.1
Average syntenic block size (Mbp)	1.3	3.1	4.7	4.9
*K*_s_ peak value	0.64	0.48	0.025	0.05
Last common ancestor (mya)	21.33–39.51	16–29.63	0.830–1.54	1.67–3.09

Note

*K*_s_ = synonymous substitutions per synonymous site.

a Total number of annotated genes in cañahua and the comparison species.

b Total number of unique syntenic genes in cañahua and the comparison species.

We calculated the rate of synonymous substitutions per synonymous site (*K*_S_) in duplicate gene pairs between cañahua and the A‐ and B‐subgenome chromosomes found in quinoa (Jarvis et al., 2017). Clear peaks are present at *K*_S_ = 0.025 and 0.05 for the A‐ and B‐subgenome comparisons, respectively, reflecting a notable difference in the estimated time since the A and B subgenomes of quinoa last shared a common ancestor with cañahua (Appendix S6). Indeed, depending on whether an *A. thaliana*–based synonymous mutation rate (Koch et al., 2000) or a core eukaryotic–based rate is used (Lynch and Conery, 2000) in the calculation, the A‐subgenome of quinoa last shared a common ancestor with cañahua approximately 0.8–1.5 mya, whereas the B‐subgenome and cañahua have been diverged for nearly twice as long (1.7–3.1 mya). *K*_S_ values suggest that the last common ancestor between cañahua and beet was approximately 16–29.6 mya, whereas the last common ancestor between cañahua and amaranth was more distant at 21.3–39.5 mya (Appendix S6, Table 4).

## DISCUSSION

The value of incorporating Hi‐C data and long reads into the assembly is clear when comparing ASRA and PGA2 assemblies. The Hi‐C data increased contiguity of PGA2 significantly by reducing the assembly from 3015 scaffolds to nine chromosome‐scale scaffolds, while the long‐read sequence dramatically reduced the number of gaps (by 75%) in the assembly as well as increasing the total assembly size. One notable disadvantage to developing a genome assembly based on short reads is the difficulty of properly assembling repetitive elements (Richards, 2018). When the read length is shorter than the repeat element, the reads collapse into single contigs, resulting in genome assemblies that can be significantly smaller than the actual size of the genome such as seen here. For example, the telomeric repeat in PGA2 was largely collapsed into a single contig that was not scaffolded to any of the chromosomes. Although there are traces of telomere sequence on several of the nine scaffolds (Fig. 1), the integrity of this element was largely lost. Nonetheless, the assembly method reported here is cost‐effective, requiring only inexpensive Illumina short‐read technology for the initial assembly and Hi‐C scaffolding, while the more expensive long reads (PacBio) necessary for gap‐filling are only needed at low coverage.

The high level of synteny between cañahua chromosomes and the A‐subgenome chromosomes of quinoa, as well as the high chloroplast similarity and *K*_S_ values, provides strong evidence supporting a New World A‐genome diploid as the maternal cytoplasm donor of the A‐subgenome in the allopolyploidization of quinoa. However, given the closer proximity between the Eurasian landmass (B‐subgenome origin) with North America versus South America, a North American A‐genome diploid donor is more logical than a South American origin donor, such as cañahua. Thus it is unlikely that cañahua is the direct ancestor of the A‐subgenome in quinoa, suggesting that future genomic analyses of the more than 45 putative A‐genome diploid *Chenopodium* species should provide important insight into the polyploidization events that underlie the evolution and domestication of the New World AABB *Chenopodium* species complex that includes free‐living *C. berlandieri* Moq. subsp. *berlandieri*, *C. quinoa* var. *melanospermum* Hunz., *C. quinoa* subsp. *milleanum* Aellen, and *C. hircinum* Schrad., along with their domesticated forms *C. quinoa* and *C. berlandieri* subsp. *nuttalliae* (Saff.) H. Dan. Wilson & Heiser (Wilson, 1990). Indeed, recently reported read‐mapping percentages reveal that *C. watsonii* A. Nelson and *C. sonorense* Benet‐Pierce & M. G. Simpson, both wild diploids collected in the southwestern United States, align more closely to the quinoa A‐subgenome than does cañahua, with *C. watsonii* exhibiting the highest mapping percentage (Jellen et al., 2019). Whole‐genome sequencing of an additional 24 putative A‐genome diploid *Chenopodium* species, originating from across North, Central, and South America, is currently underway in our laboratory.

Careful evaluation of chromosomes within the Amaranthaceae family can shed light on how these genomes evolved over time and what role structural changes have played in biological function. For example, homologs of Cp5 are highly conserved in both the A and B subgenomes of quinoa (Cq5A and Cq5B), but there is clear structural variation in comparison to the homolog in beet, Bv5 (Fig. 4A). One of the amaranth homologs of Cp5 is collinear (Ah2), whereas the second homolog is split between two chromosomes (Ah11 and Ah12) but also reflects a similar order. This may be evidence that a terminal inversion occurred in the evolution of beet after the divergence from a common ancestor. Homologs of Cp9 also show an evolutionarily interesting pattern. Whereas Cp9 is conserved in the A‐subgenome of quinoa (Cq4A), demonstrated both by a syntenic dot plot (Fig. 4A) and a high number of syntenic genes (1323; Appendix S5), the B‐subgenome homolog has a much different structure and less than half the number of syntenic genes (536). Meanwhile, beet and amaranth both have unique rearrangements of this homolog (Bv9 and Ah1, respectively), suggesting that the order of genes along this molecule may not hold significant biological importance.

In conclusion, the reference‐quality, chromosome‐scale assembly of cañahua presented here dramatically improves the existing resources for this regionally important subsistence crop. The reference genome provides a critical genomic tool needed to draw attention to cañahua, which should lead to renewed interest in improved varietal development using modern plant breeding techniques, including marker‐assisted selection and genomic selection (Jannink et al., 2010; Brachi et al., 2011). The genome annotation reported here will undoubtedly facilitate gene discovery efforts within the species, allowing researchers to move quickly from genetic linkage/linkage disequilibrium experiments to possible candidate gene targets. Indeed, once target genomic regions are identified, enhanced marker‐assisted breeding methods can be more effectively employed. Given that the only other domesticated *Chenopodium* species are complex allotetraploids (*C. quinoa* and *C. berlandieri* subsp. *nuttalliae*), we anticipate that cañahua will serve as a simplified genetic model for the family (Bertioli et al., 2016; Du et al., 2018). Lastly, the resequencing information presented here for a large diversity panel of cañahua accessions collected from across the Altiplano should provide the preliminary data needed for germplasm conservation and core‐collection development.

## Supporting information


**APPENDIX S1.** Outline of the genome assembly process.

**APPENDIX S2.** Length and contig number for each chromosome‐scale scaffold in PGA2.

**APPENDIX S3.** Repetitive element classification for final assembly (PGA2) as reported by RepeatMasker.

**APPENDIX S4.** Orthologous genes were identified between cañahua and beet (A), amaranth (B), and quinoa (C) to detect orthologous chromosome relationships.

**APPENDIX S5.** Comparison of gene synteny between cañahua and the two subgenomes of quinoa.

**APPENDIX S6.** Rate of synonymous substitutions per synonymous site (*K*_s_). *K*_s_ values within duplicated gene pairs between cañahua with amaranth (red), beet (yellow‐brown), tetraploid quinoa (green), the A‐subgenome of quinoa (blue), and the B‐subgenome of quinoa (purple).

## Data Availability

The raw sequences are deposited in the National Center for Biotechnology Information (NCBI) Sequence Read Archive database under the BioProject ID PRJNA552289 with the following accession numbers: SRR9661228 (PacBio), SRR9661229 (Hi‐C), and SRR9640731–SRR9640759 and SRR9620980 (resequencing panel). Bulk data downloads, including annotations and BLAST analysis, and JBrowse viewing of the final proximity‐guided assembly are available at CoGe (https://genomevolution.org/coge/; Genome id53872).
