# Supplementary material for: The genome of Chenopodium pallidicaule: An emerging Andean super grain
Source: Appl Plant Sci. 2019 Nov 8;7(11):e11300. doi: 10.1002/aps3.11300 (PMC6858295; doi:10.1002/aps3.11300)

DOI

APPENDIX S1. Outline of the genome assembly process. The ALLPATHS-LG assembler was used to develop an initial assembly, named the ALLPATHS-LG short-read assembly (ASRA), from Illumina short reads. The first proximity-guided assembly was performed using Hi-C data and the Proximo pipeline (PGA1), diagrammed in the bottom left of the flowchart. Overlapping chromatin was formalin-fixed, the genome was fragmented, and fixed fragments were then selected and circularized. Illumina reads were generated, and forward and reverse reads were aligned to the ASRA scaffolds. Crosslink frequency was used to first group, then order, and then orient the scaffolds along pseudochromosomes. Proximity-guided assembly was followed by gap-filling with PacBio long reads (PBJelly), as demonstrated in the top center, and genome polishing by Arrow and Pilon (PGA1.5). PGA1.5 was broken at all N-gaps and areas of low PacBio read coverage (PolarStar), then underwent a second round of proximity-guided assembly (PGA2). A comparison of PGA1 and PGA2 is shown in the bottom right of the diagram, where increasing frequency of cross-linking is illustrated by increasing color intensity.

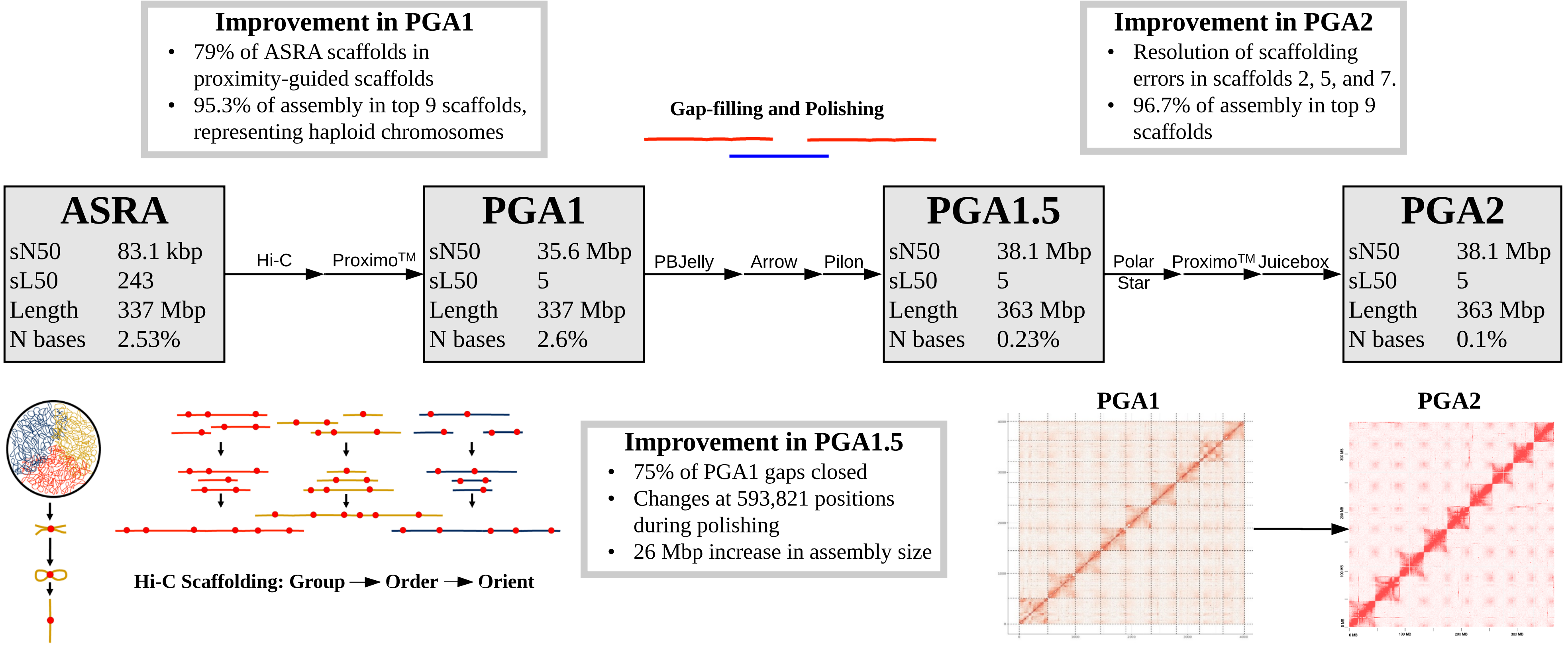

Supplement: Supplementary file 1 — APPENDIX S1. Outline of the genome assembly process. [file APS3-7-e11300-s001.pdf]
