# Supplementary material for: The genome of Chenopodium pallidicaule: An emerging Andean super grain
Source: Appl Plant Sci. 2019 Nov 8;7(11):e11300. doi: 10.1002/aps3.11300 (PMC6858295; doi:10.1002/aps3.11300)
Supplement: Supplementary file 2 — APPENDIX S2. Length and contig number for each chromosome‐scale scaffold in PGA2. [file APS3-7-e11300-s002.docx]

**APPENDIX S2.** Length and contig number for each chromosome-scale scaffold in PGA2.

| **Scaffold name** | **Contigs** | **Length (Mbp)** | **Percentage of final assembly** |
| --- | --- | --- | --- |
| Cp1 | 366 | 37.93 | 10.5% |
| Cp2 | 376 | 35.65 | 9.8% |
| Cp3 | 347 | 38.12 | 10.5% |
| Cp4 | 413 | 39.85 | 11.0% |
| Cp5 | 474 | 45.40 | 12.5% |
| Cp6 | 423 | 41.46 | 11.4% |
| Cp7 | 376 | 35.49 | 9.8% |
| Cp8 | 480 | 40.69 | 11.2% |
| Cp9 | 331 | 33.52 | 9.2% |
| Unassigned contigs | 4,632 | 14.40 | 4.0% |
| Total | 8218 | 362.51 | 100% |
