# Supplementary material for: The genome of Chenopodium pallidicaule: An emerging Andean super grain
Source: Appl Plant Sci. 2019 Nov 8;7(11):e11300. doi: 10.1002/aps3.11300 (PMC6858295; doi:10.1002/aps3.11300)
Supplement: Supplementary file 3 — APPENDIX S3. Repetitive element classification for final assembly (PGA2) as reported by RepeatMasker. [file APS3-7-e11300-s003.docx]

**APPENDIX S3.** Repetitive element classification for final assembly (PGA2) as reported by RepeatMasker.

| **Repeat class** | **Repeat name** | **Count** | **bp Masked** | **% Masked** |
| --- | --- | --- | --- | --- |
| DNA |  | 1937 | 393,444 | 0.11% |
|  | CMC-EnSpm | 16,383 | 8,608,444 | 2.38% |
|  | MULE-MuDR | 4740 | 2,120,134 | 0.59% |
|  | MuLE-MuDR | 5987 | 6,196,185 | 1.71% |
|  | PIF-Harbinger | 2938 | 1,474,098 | 0.41% |
|  | PiggyBac | 29 | 5974 | 0.00% |
|  | TcMar-Mogwai | 400 | 390,325 | 0.11% |
|  | TcMar-Stowaway | 12,979 | 2,472,544 | 0.68% |
|  | Zisupton | 175 | 71,505 | 0.02% |
|  | hAT | 923 | 203,143 | 0.06% |
|  | hAT-Ac | 13,999 | 5,806,490 | 1.60% |
|  | hAT-Blackjack | 178 | 69,670 | 0.02% |
|  | hAT-Charlie | 94 | 16,929 | 0.00% |
|  | hAT-Tag1 | 2141 | 344,649 | 0.10% |
|  | hAT-Tip100 | 880 | 238,523 | 0.07% |
| LINE |  | — | — | — |
|  | CRE-II | 185 | 162,070 | 0.04% |
|  | I | 154 | 67,059 | 0.02% |
|  | I-Jockey | 726 | 219,931 | 0.06% |
|  | L1 | 5259 | 2,678,497 | 0.74% |
|  | R1 | 69 | 34,818 | 0.01% |
|  | RTE-BovB | 3040 | 818,549 | 0.23% |
| LTR |  | 175 | 37,749 | 0.01% |
|  | Cassandra | 509 | 87,706 | 0.02% |
|  | Caulimovirus | 133 | 222,562 | 0.06% |
|  | Copia | 24,683 | 29,530,512 | 8.16% |
|  | ERV1 | 81 | 10,177 | 0.00% |
|  | Gypsy | 49,562 | 68,205,239 | 18.84% |
|  | Ngaro | 92 | 8530 | 0.00% |
| RC |  | — | — | — |
|  | Helitron | 1956 | 553,770 | 0.15% |
| Unknown |  | 193,885 | 57,981,971 | 16.01% |
| Total interspersed |  | 344,292 | 189,031,197 | 52.21% |

| Low complexity |  | 17,678 | 933,652 | 0.26% |
| --- | --- | --- | --- | --- |
| Satellite |  | 1575 | 926,586 | 0.26% |
| 5S |  | 848 | 96,514 | 0.03% |
| Simple repeat^a^ |  | 99,744 | 5,987,414 | 1.65% |
| rRNA |  | 219 | 32,987 | 0.01% |
| Total |  | 464,356 | 197,008,350 | 54.41% |

*Note:* LINE = long interspersed nuclear elements; LTR = long terminal repeat; RC = Rolling circle.

^a^The most common mono-, di-, tri-, and tetranucleotide repeat motifs were (T)_n_, (TA)_n_, (ATT)_n_, and (TTTA)_n_, respectively.
