# Supplementary material for: The genome of Chenopodium pallidicaule: An emerging Andean super grain
Source: Appl Plant Sci. 2019 Nov 8;7(11):e11300. doi: 10.1002/aps3.11300 (PMC6858295; doi:10.1002/aps3.11300)
Supplement: Supplementary file 4 — APPENDIX S4. Orthologous genes were identified between cañahua and beet (A), amaranth (B), and quinoa (C) to detect orthologous chromosome relationships. [file APS3-7-e11300-s004.pdf]

APPENDIX S4. Orthologous genes were identified between cañahua and beet (A), amaranth (B), and quinoa (C) to detect orthologous chromosome relationships. Cañahua chromosomes are shown on the vertical axis with the comparison species on the horizontal axis. Total syntenic bases are shown between all chromosome comparisons. Syntenic relationships are shown in red and transition to white as the amount of synteny decreases.

A. Cañahua and beet (*B. vulgaris*)

	Cp1	Cp2	Cp3	Cp4	Cp5	Cp6	Cp7	Cp8	Cp9
**Bv1**	28325211	0	0	0	0	0	0	0	0
**Bv2**	0	32294663	0	0	0	0	0	0	613860
**Bv3**	0	0	30553938	0	0	0	0	0	0
**Bv4**	0	0	0	26001022	0	0	0	0	0
**Bv5**	0	0	0	0	32396310	0	0	0	0
**Bv6**	0	0	0	0	0	33763996	0	0	410607
**Bv7**	0	0	187090	0	0	0	29222897	0	0
**Bv8**	0	0	0	0	0	0	0	31233495	0
**Bv9**	1986690	0	0	0	0	0	0	0	24113218

B. Cañahua and amaranth (*A. hypochondriacus*)

**Ah5**	23589839	0	0	0	0	0	0	0	0
**Ah10**	274635	20375526	0	191909	0	0	0	0	0
**Ah13**	0	0	24075906	0	0	0	0	0	0
**Ah6**	0	0	0	24079690	0	0	0	0	0
**Ah12**	0	6043249	0	0	15391364	0	0	0	0
**Ah3**	405633	0	0	0	0	28013455	0	0	0
**Ah8**	0	0	0	0	0	0	28710679	0	0
**Ah14**	0	0	0	0	0	0	0	31339657	0
**Ah1**	2031098	0	0	0	0	0	0	0	46011382
**Ah11**	7909746	2943533	0	0	18699421	0	0	0	0
**Ah16**	728985	12811486	0	0	0	0	0	0	0
**Ah7**	0	0	10581410	0	5515624	0	0	0	0
**Ah4**	0	0	16807875	24046181	0	0	0	0	0
**Ah2**	0	0	0	890770	18552821	26295005	0	0	0
**Ah15**	0	0	747922	0	0	0	20248458	0	0
**Ah9**	0	0	0	0	0	0	0	28879551	0

C. Cañahua and quinoa (*C. quinoa*)

**Cq1A**	28395535	0	0	0	0	0	0	0	0
**Cq2A**	0	28696603	0	0	0	0	0	0	0
**Cq3A**	0	0	32396029	0	0	0	0	0	0
**Cq4A**	0	435499	0	28373673	0	0	0	0	0
**Cq5A**	0	0	0	0	40175907	0	0	0	132648
**Cq6A**	0	0	0	0	0	35717891	0	0	441719
**Cq7A**	0	0	1159408	0	0	0	29575796	0	0
**Cq8A**	0	0	0	0	0	0	0	24327900	0
**Cq9A**	1270829	0	0	0	0	0	0	55572	28592795
**Cq1B**	23457050	4524204	0	0	0	0	0	0	0
**Cq2B**	6555868	23589150	0	1426684	0	0	0	0	0
**Cq3B**	0	0	32068509	0	0	0	0	0	0
**Cq4B**	0	0	0	27936159	0	0	0	0	0
**Cq5B**	0	0	0	0	37134286	0	0	0	0
**Cq6B**	0	0	0	0	0	29941194	0	0	0
**Cq7B**	0	0	0	0	0	0	22118319	0	8681948
**Cq8B**	0	0	0	0	0	0	0	29349842	0
**Cq9B**	1218908	0	0	0	0	0	3388586	0	16734715
