# Supplementary material for: The genome of Chenopodium pallidicaule: An emerging Andean super grain
Source: Appl Plant Sci. 2019 Nov 8;7(11):e11300. doi: 10.1002/aps3.11300 (PMC6858295; doi:10.1002/aps3.11300)
Supplement: Supplementary file 5 — APPENDIX S5. Comparison of gene synteny between cañahua and the two subgenomes of quinoa. [file APS3-7-e11300-s005.docx]

**APPENDIX S5.** Comparison of gene synteny between cañahua and the two subgenomes of quinoa.

| **Quinoa chromosome** | **Syntenic blocks** | **Syntenic gene pairs** | **Syntenic gene pairs/block** |
| --- | --- | --- | --- |
| Cq1A | 28 | 1364 | 48.7 |
| Cq1B | 22 | 807 | 36.7 |
| Cq2A | 26 | 1355 | 52.1 |
| Cq2B | 26 | 938 | 36.1 |
| Cq3A | 24 | 1522 | 63.4 |
| Cq3B | 30 | 1473 | 49.1 |
| Cq4A | 18 | 1376 | 76.4 |
| Cq4B | 20 | 1416 | 70.8 |
| Cq5A | 26 | 1742 | 67.0 |
| Cq5B | 26 | 1662 | 63.9 |
| Cq6A | 30 | 1661 | 55.4 |
| Cq6B | 28 | 1540 | 55.0 |
| Cq7A | 16 | 1383 | 86.4 |
| Cq7B | 24 | 665 | 27.7 |
| Cq8A | 14 | 1379 | 98.5 |
| Cq8B | 30 | 1316 | 43.9 |
| Cq9A | 14 | 1291 | 92.2 |
| Cq9B | 16 | 520 | 32.5 |
| A-Subgenome Total | 196 | 13,073 | 71.1 |
| B-Subgenome Total | 222 | 10,337 | 46.2 |
