# Supplementary material for: The genome of Chenopodium pallidicaule: An emerging Andean super grain
Source: Appl Plant Sci. 2019 Nov 8;7(11):e11300. doi: 10.1002/aps3.11300 (PMC6858295; doi:10.1002/aps3.11300)

DOI

APPENDIX S6. Rate of synonymous substitutions per synonymous site (*K*s). *K*s values within duplicated gene pairs between cañahua with amaranth (red), beet (yellow-brown), tetraploid quinoa (green), the A-subgenome of quinoa (blue), and the B-subgenome of quinoa (purple).

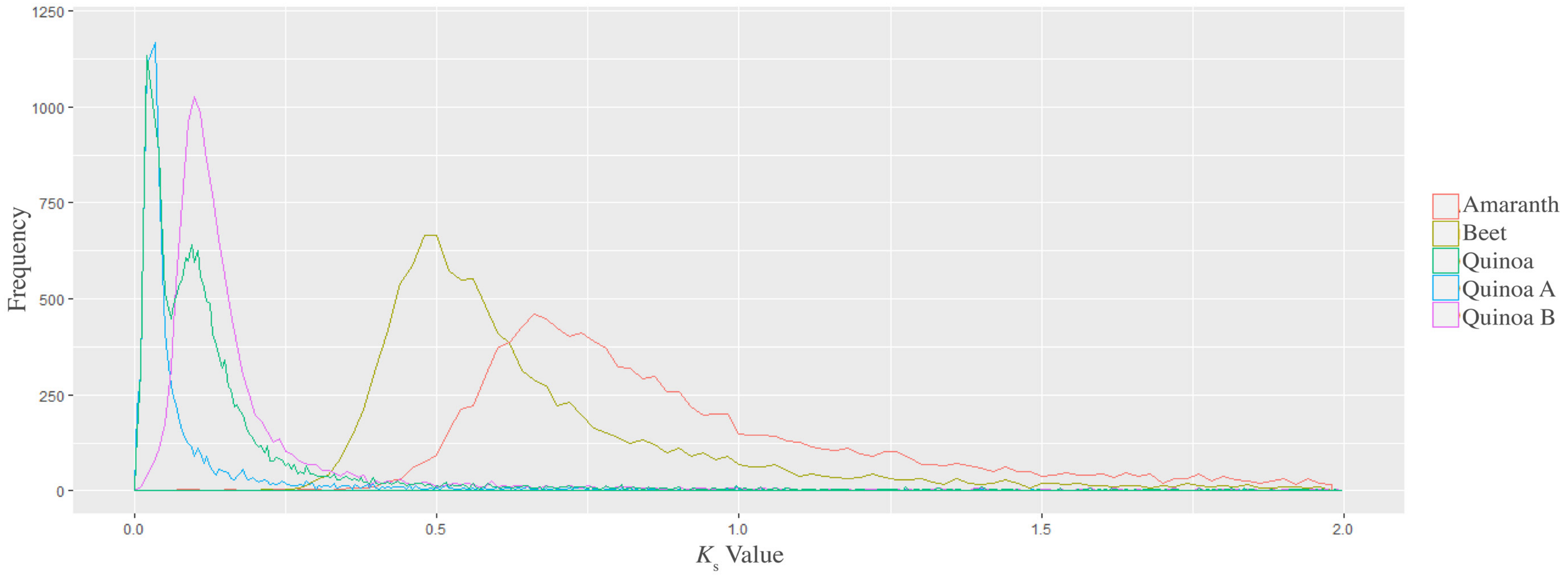

Supplement: Supplementary file 6 — APPENDIX S6. Rate of synonymous substitutions per synonymous site (K s). K s values within duplicated gene pairs between cañahua with amaranth (red), beet (yellow‐brown), tetraploid quinoa (green), the A‐subgenome of quinoa (blue), and the B‐subgenome of quinoa (purple). [file APS3-7-e11300-s006.pdf]
